# The Diverse Landscape of Fascial Manual Medicine

**DOI:** 10.7759/cureus.102052

**Published:** 2026-01-22

**Authors:** Bruno Bordoni

**Affiliations:** 1 Physical Medicine and Rehabilitation, Foundation Don Carlo Gnocchi, Milan, ITA

**Keywords:** chiropractic, collagen, fascia, fascial, fascintegrity, manual therapy, myofascial, osteopathic manipulative

## Abstract

Manual medicine is a clinical approach where patients are administered non-invasive, non-pharmacological techniques to address chronic or acute conditions, such as osteopathy, chiropractic, and physiotherapy. The effect is always systemic, even though the chosen manual strategy may focus on a specific area of ​​the body, just as with surgery and pharmacology. Fascia is a subject of intense debate among manual medicine and clinical practitioners, with its definition and terminology still lacking a universal consensus. Manual medicine does not consider fascial continuity as segments or layers, nor does it differentiate fibrous tissue from non-fibrous tissue. While a clinician may target a viscus, muscle, or bone, a manual medicine practitioner's technique begins at the epidermis and never penetrates the skin. This article provides a brief review of the history and various major international currents of thought on the topic of fascia, including its nomenclature and function. It reiterates and compares the view of fascia from the perspectives of manual medicine and our study group, the Foundation of Osteopathic Research and Clinical Endorsement (FORCE).

## Editorial

Manual medicine views the body as a functionally integrated, interdependent unit rather than a collection of separate layers, where neural, endocrine, immune, and psychological mechanisms operate in unison. The human body is not a machine, but a living entity that manifests a multi-layered organization, ranging from the macroscopic level down to microscopic and quantum foundations. Tissues are studied in detail using microscopic images; this concept began with the discovery of the microscope, and with increasingly advanced technology, leading to three-dimensional images of cellular complexes (holographic flow cyto-tomography, diffusion tensor magnetic resonance imaging). Quantum biology is advancing rapidly, driven by Lambert and colleagues' assertion that “Every chemical process relies on quantum mechanics”. According to the most recent scientific literature, all chemical reactions that are the basis of cellular life reflect the laws of mechanical physics or quantum physics. For instance, electron transport between protein structures, often occurring independently of distance, is facilitated by the quantum mechanical process of tunneling. This phenomenon does not require energy; however, it is capable of regulating the metabolism of cells, tissues, and the human body. Quantum biology has fostered a new era of nanotechnology that is capable of observing life at the atomic level (using scanning tunneling microscopes). This is not science fiction, but technological and scientific progress [1].

Bodily activation in response to a stimulus will have a systemic response, even if the stimulus is localized. This stimulus at the macroscopic system triggers a response at both the macroscopic and microscopic levels. Furthermore, nanoscopic or quantum mechanisms enable every cell in the body to communicate with all others. For instance, a gentle touch on the skin will activate low-threshold cutaneous receptors (C-tactile afferents), sending proprioceptive signals along the spinothalamic pathways, stimulating the limbic area and the cortical areas for the management of sensory signals, influencing behavior and movement. Thus, a gentle touch, such as a caress, will stimulate the production of hormones such as oxytocin and neuropeptides deriving from the opioid system. The resulting hormonal response will affect parasympathetic neurometabolism, reducing the heart rate, raising the pain threshold, and improving emotional and motor status [1].

When the cell changes shape and function, the electrons change their charge and start moving, generating vibrations (biophonons and biophotons). These vibrations generate new electromagnetic fields, which expand from the cell itself, creating electromagnetic waves. Electromagnetic waves received by cellular components (e.g., DNA) can alter their structural architecture, inducing further electromagnetic field generation and maintaining a continuous information exchange. As Dr. Kim states, every biochemical action is linked to the law of quantum physics, which has no boundaries [1]. Cells can also influence each other through electromagnetic fields/waves in the absence of direct contact. An example is ephaptic transmission, where synaptic junctions and myelin tissue are activated remotely through the phenomenon of quantum tunneling. Ions, protons, electrons, and various atoms can cross a membrane (carried by electromagnetic waves), regardless of the electromagnetic potential of the membrane itself. These quantum particles can cross any membrane and promote depolarization, creating a response at the microscopic and then macroscopic levels. Quantum tunneling is a biological strategy in which information is transmitted between a nearby or distant cell, without the need to produce energy for the transmission. These mechanisms can explain the pharmacological action or simply the causes of referred pain, where the clinical picture is not always linear with the symptoms [2]. 

According to the thinking of the Foundation of Osteopathic Research and Clinical Endorsement (FORCE) research group, the macro-micro-nanoscopic and functional unity vision of the human organism influences how the fascia or fascial continuum is conceived in manual medicine, both from the point of view of nomenclature and function [1,3]. Manual medicine takes the topic of fascia into extreme consideration, as manual approaches focus on the mobilization of this tissue to obtain systemic responses to improve symptoms through multiple different techniques.

This article briefly reviews the history and various major international currents of thought on the topic of fascia, including its nomenclature and function, reiterating and comparing the view of fascia from the perspective of manual medicine.

Origins of fascia

**Figure 1:**
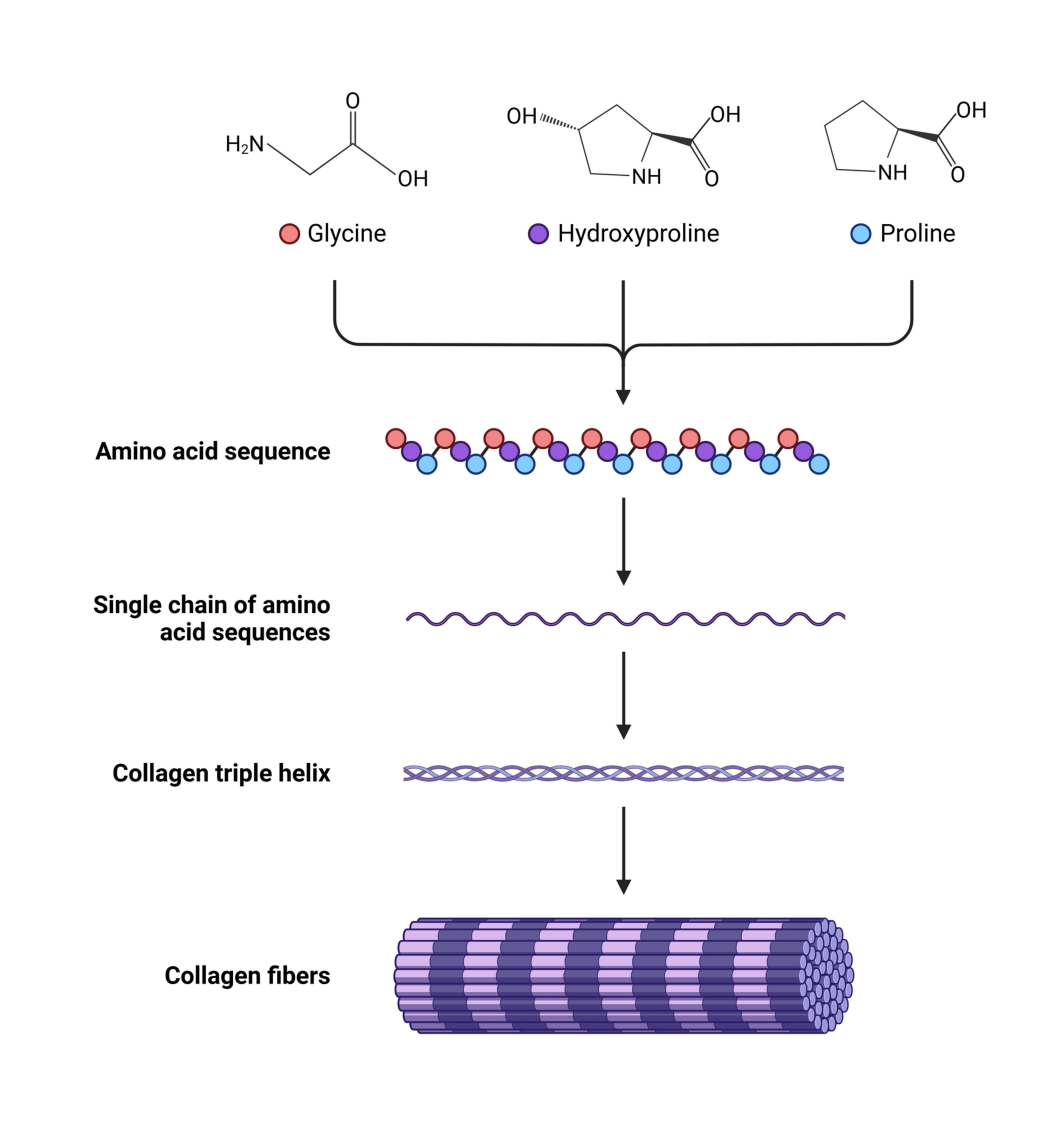
Schematic illustration of the structure of collagen starting from the constituting amino acids Created in BioRender. Bordoni, B. (2026) https://BioRender.com/h1gzs52

History explains the present by illuminating the steps that shaped our current reality. After the Egyptians, who identified an elastic tissue during mummification work, the Persians were the next scholars to introduce the concept of fascial tissue or fascia (7th-13th century AD). The books of Persian anatomists were rediscovered by European scholars from the sixteenth century. The term fascia was reintroduced in 1615 by Dr. Crooke and was shortly thereafter used by Dr. Cooper, who employed the term fasces [3]. In 1780, the fascia was described from a microscopic point of view for the first time by Dr. Simmons, highlighting the fascia as a network with water drops. In 1802, Dr. Bichat established the foundational definition of fascia that aligns with 21st-century anatomical understanding, characterizing it as a systemic fibrous tissue present throughout the human body (Figure 1). In 1804, the epidermis was excluded from the concept of fascial tissue by Dr. Cooper [4].

Throughout the 19th century, the term 'fascia' appeared frequently in medical, surgical, and anatomical literature, with various names attributed to the tissue based on its location or depth [3,4]. In 1851, Dr. Wilson defined fascia as beginning from the dermis and excluding the epidermis, a concept subsequently adopted in the 1858 edition of Gray’s Anatomy [3]. In 1935, Dr. Singer reaffirmed that the fascia is a fibrous membrane that surrounds the major body structures and the skeletal muscles [4].

During the late 20th century, groups of anatomists sought to improve the conceptualization and nomenclature of fascia, notably through the anatomical terminology of the Federative Committee on Anatomical Terminology (FCAT), and the International Federation of Associations of Anatomists in 1998 [3,4]. Also included is the Federative International Programme for Anatomical Terminology (FIPAT), a component of the International Federation of Associations of Anatomists (IFAA) [4].

In the 21st century, two groups of researchers tried to better discern the concept of how fascial tissue can be classified. Towards this aim, the Fascia Research Society (FRS) was created in 2007, within which we find the Committee for Fascial Nomenclature, while FORCE was created in 2013 [3].

From the anatomist's perspective

Anatomists define fascia as a dissectible connective tissue that wraps, separates, and supports muscles and organs. In this context, anatomy is understood as the study of physical structure through both the intellectual analysis and the physical dissection of cadavers. Anatomy studies focus on structure, and not function. The classification of fascia is restricted solely to fibrous structures characterized by a dense, irregular connective tissue composition [4]. Anatomists recognize fascial layers as fascia of muscles, intermuscular septum, retinaculum tendinis, parietal fascia, tela subserosa parietalis, visceral fascia, tela subserosa visceralis, extraserosal fascia, and neurovascular sheath [4]. Knowledge of the depth of the different anatomical layers is vital for the surgeon to ensure patient health, avoid iatrogenic damage, and target the tissue of interest of the surgical procedure as specifically as possible.

The definition of fascia has excluded the epidermis and been limited to fibrous connective tissue since the early 19th century (specifically 1802-1804). Knowing the position of the fascial layers is educationally useful, but not essential for manual medicine. The classification of what can be understood as fascia by anatomists is not useful to a clinician using manual medicine. According to anatomists, not all anatomical components must necessarily be connected [4]. Every cell is in constant communication with its environment and neighboring cells [1,3]. Another barrier between anatomists and manual therapists is their differing definitions of connective tissue. The muscle tendon, for example, is not recognized as fascial tissue, despite being connective tissue [4]. Why has this division persisted for centuries? From the FORCE perspective, such a separation is practically nonsensical. When performing an action like bending the elbow, the entire body participates, not just the arm. The movement continues seamlessly because tendons are treated as a distinct structural layer, separating the muscle-bone connection from the surrounding neurological, muscular, and articular systems. Lately, anatomists have criticized the choices of nomenclature and meaning of what can be described as fascia, compared to the FRS group [4].

From the orthopedic perspective

The FRS research group, which includes the Stecco family, is primarily composed of orthopedic surgeons. Orthopedic surgeons must understand the layers, as this is useful not only for diagnosis and any subsequent surgical intervention in the selected area, but also for invasive pharmacological approaches, such as joint injections.

**Figure 2:**
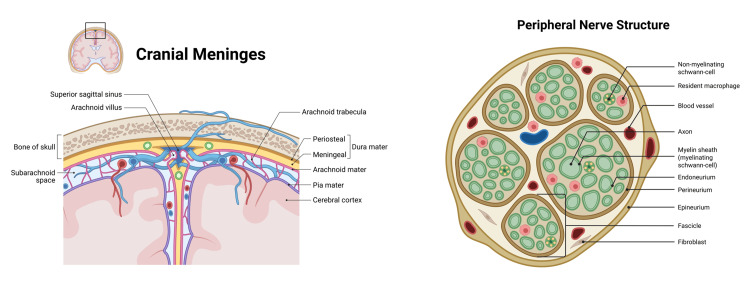
The image on the left illustrates the various meningeal layers of the skull, considered as fascia by the Fascia Research Society, as well as the different fascial neural layers of the peripheral nerve Created in BioRender. Bordoni, B. (2026) https://BioRender.com/h1gzs52

Experts have highlighted inconsistencies in how the FRS describes fascia, particularly regarding the distinction between anatomical structure and physiological function [4]. FRS divides fascia into four organs: superficial fascia, deep musculoskeletal fascia, visceral fascia, and neural fascia [5]. For anatomists, fascia is not an organ; however, every cell of every tissue produces substances emitted in autocrine and paracrine modes [1,3]. FORCE is in line with FRS, which recognizes adipose tissue, dermis, membranous subcutaneous tissue, muscular and visceral ligaments and tendons, as well as joints, periosteum, mesentery, and the tissues covering the purely neural tissue as fascia (Figure 2) [4,5]. Unlike the FRS distinction, anatomists often treat anatomical and physiological systems as synonymous, blurring the line between structure and function [4]. Furthermore, for anatomists, there is an incongruity in defining the fascial system as an anatomical system, since the former is already within the latter [4].

Compared to the orthopedic surgeon's view, manual medicine has no practical interest in performing techniques or knowing all the various fascial layers [1,3]. On the one hand, without an instrumental examination (for example an ultrasound), the hand is not able to recognize the difference between layers such as the endomysium, the perimysium or the epimysium, and on the other hand, knowing that the human body acts as a unit/organism, by placing the hand on the epidermis (with different pressures), all the layers are involved in adapting to the pressure generated by the operator [1,3]. Unlike anatomists and orthopedists, manual medicine practitioners do not work on cadavers or in the operating room.

From the perspective of manual medicine

For manual medicine, the body is not a machine that can be divided into layers, but rather an organism whose function is an indivisible set of systems. From the perspective of FORCE, which represents manual medicine, it makes no practical sense to work manually on the patient, viewing them as compartments [1,3]. The fundamental principle of the body is that every component is interconnected, making the whole present in every part. Fibroblasts and telocytes are present throughout the body, transcending conventional anatomical layering. This necessitates a 3D, rather than 2D, approach to understanding their distribution and role. By doing this, one realizes that cells are often branched, involve several different layers, and can create, by necessity, more *de novo* and temporary branches (filopodia and lamellipodia), and communicate with each layer (microRNA, transport vesicles, and more) [1,3].

From the FORCE perspective, fascia refers to connective tissue. In every anatomy book, when we look for connective tissue, we highlight specialized connective tissue, which is recognized as fascia by our research group's classification [1,3]. As a clinician who uses manual medicine and works with the whole body, one of the major differences that sets us apart from anatomists and orthopedists is the distinction and inclusion of solid fascia (such as bones, cartilage, and others) and fluid fascia (such as body fluids). One of the important reasons that allows us to include different body components as fascial tissue is its embryological origin [1,3]. Fascial tissue derives from the mesoderm and the ectoderm, as previously described in detail in other articles, where the reader is invited to delve deeper [1,3]. For example, the fascia of the skull and part of the neck and shoulders derive from the ectoderm, which merges seamlessly with the fascia of mesodermal origin. The neck/shoulder muscles themselves have a dual phylogenetic origin [1]. Based on this concept, the FORCE perspective suggests that tissues derived from both the ectoderm and mesoderm can be classified as fascial tissue. Anatomists emphasize that embryology is essential for understanding function and, consequently, hormesis. However, the ontological origin of tissues is frequently overlooked when classifying what constitutes fascia.

**Figure 3:**
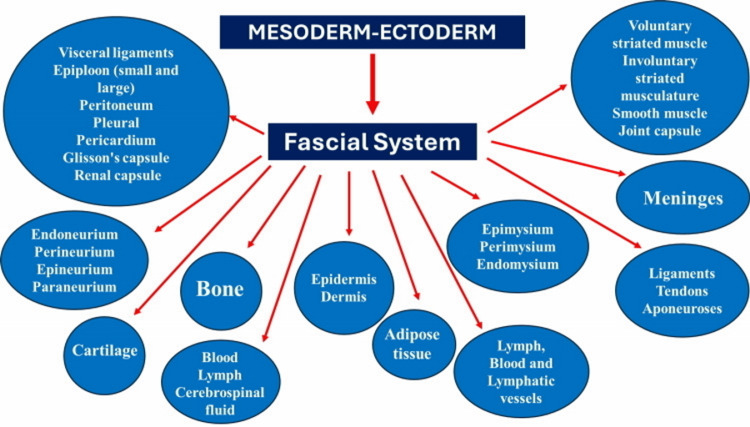
The image schematizes the body components that the FORCE research group considers and includes in the concept of fascia Source: Bordoni et al [1], used under the terms of the Creative Commons Attribution License CC-BY 4.0.

FORCE reiterates the concept of what can be considered as fascia in manual medicine. Briefly, the fascial continuum is the result of the evolution of the perfect synergy among different tissues, liquids, and solids, capable of supporting, dividing, penetrating, feeding, and connecting all the districts of the body: epidermis, dermis, fat, blood, lymph, blood, and lymphatic vessels, cerebrospinal fluid, the tissue covering the nervous filaments (endoneurium, perineurium, epineurium, paraneurium), voluntary striated muscle fibers and the tissue covering and permeating it (epimysium, perimysium, endomysium), ligaments, tendons, aponeurosis, cartilage, bones, joint capsule, meninges, involuntary striated musculature and smooth muscle (all viscera derived from the mesoderm), visceral ligaments, epiploon (small and large), peritoneum, pleura, pericardium, Glisson’s capsule, and kidney capsule. The continuum constantly transmits and receives mechano-metabolic information that can influence the shape and function of the entire body (Figure 3)[1].

Research is an endless journey of discovery, not a final destination, as knowledge is meant to be explored rather than fully constrained. Confidence in science must not become an insurmountable constraint, but a force to continually drive intellectual curiosity forward. The data derived from knowledge should not be considered merely as anchors, with which to stop and protect one's ability to think, but should be considered as slingshots, trying to discover what lies beyond the horizon of knowledge. Science does not seek its own "reason", but the ability to understand.

The definition of fascia is a highly debated topic in literature, with different interpretations among clinical practitioners. Unlike orthopedists and anatomists, who view the human body as a collection of layers, whether continuous or discontinuous, manual medicine considers the body not as a machine and compartments, but as an organism, where everything works in unison and cannot be separated from clinical practice. This article briefly describes the differing perspectives that arise from different practitioners in conceiving fascia, as well as the fascia model of the FORCE research group. Research must make further efforts to unify the nomenclature and function of the fascial continuum.
